# Brain Atrophy Following Deep Brain Stimulation: Management of a Moving Target

**DOI:** 10.5334/tohm.546

**Published:** 2020-10-21

**Authors:** Shannon Y. Chiu, Wissam Deeb, Pamela Zeilman, Adolfo Ramirez-Zamora, Addie Patterson, Bhavana Patel, Kelly D. Foote, Michael S. Okun, Amar Patel, Leonardo Almeida

**Affiliations:** 1 Department of Neurology, Norman Fixel Institute for Neurological Diseases, University of Florida, Gainesville, FL, US; 2 Department of Neurosurgery, Norman Fixel Institute for Neurological Diseases, University of Florida, Gainesville, FL, US; 3 Department of Neurology, Yale school of Medicine, Yale University, New Haven, CT, US

**Keywords:** deep brain stimulation (DBS), ventralis intermedius nucleus (VIM), essential tremor (ET), lead shift, brain atrophy

## Abstract

**Clinical vignette:**

A 51-year-old man with essential tremor (ET) had bilateral ventralis intermedius nucleus deep brain stimulation (VIM-DBS) placed to address refractory tremor. Despite well-placed DBS leads and adequate tremor response, he subsequently experienced worsening. Re-programming of the device and reconfirming the electrical thresholds for benefits and side effects were both performed. Six years following DBS implantation, repeat imaging revealed brain atrophy and a measured lead position change with a coincident change in clinical response.

**Clinical dilemma:**

What do we know about brain atrophy affecting lead placement and long-term DBS effectiveness? What are the potential strategies to combat narrowed therapeutic thresholds and to maximize DBS therapeutic benefit?

**Clinical solution:**

Decreasing the electrical field of stimulation and programming in a bipolar configuration are strategies to provide symptomatic tremor control and to minimize stimulation-induced side effects.

**Gaps in knowledge:**

Currently, effects of brain atrophy, and factors underpinning emergence of side effects and/or loss of benefit in chronic VIM-DBS remain largely unexplored.

## Clinical vignette

A 51-year-old right-handed man with bipolar affective disorder, and a longstanding history of essential tremor (ET), underwent bilateral ventralis intermedius nucleus deep brain stimulation (VIM-DBS) at the University of Florida (UF) to address refractory tremor. He failed trials of maximally tolerated dosages of propranolol, primidone, and topiramate. He reported a strong family history of tremor, which affected his father, paternal grandmother, and great grandfather. His DBS surgeries were staged months apart with his dominant hand operated first, following our institution’s implantation and surgical protocol [1]. He had an excellent response to his left VIM-DBS at age 44. He proceeded with contralateral VIM-DBS 10 months later, however, due to medical problems unrelated to his surgery, he delayed placement of the battery and device activation. These medical issues included pneumonia and pancreatitis, as well as a stroke affecting his speech. He recovered without residual deficits, though no further details of this ischemic event could be obtained. He was lost to follow-up for 5 years, without routine programming visits or annual postsurgical neuropsychological evaluation as part of our institution’s standard of care. His right VIM-DBS lead also was not initially connected to an implantable pulse generator (IPG).

The patient eventually returned for battery placement (i.e., connected to his initially implanted left VIM-DBS), due to worsening tremors and suspected battery depletion. He underwent placement of a new dual-channel IPG, connecting the existing left DBS and the new right VIM-DBS extension(s). Notably, when his bilateral VIM-DBS leads were activated, he experienced immediate worsening of speech, bilateral arm and leg heaviness, and difficulty walking. These symptoms resolved when the DBS was inactivated, suggesting a stimulation-induced etiology.

During the monopolar electrical threshold review to determine benefits and side effects of his right VIM-DBS, he experienced side effects at very low voltages (≤2V across all four contacts). These side effects included transient sensory phenomenona and persistent motor side effects. Despite narrow thresholds for side effects, he achieved marked tremor suppression (Table 1). A monopolar review on his left VIM-DBS was repeated, demonstrating low voltages associated with side effects (Table 1). He could no longer tolerate his prior optimized left VIM-DBS setting, and experienced persistent sensorimotor side effects at now significantly lower voltages with bilateral lead activation (1.6V–1.7V).

**Table 1:** **Comparison between initial vs. current monopolar reviews, tremor scales, and brain atrophy measurements.** Table 1 shows the patient’s narrowed threshold for sensorimotor side effects on monopolar reviews over time. While patient has sustained tremor benefit from stimulation (as depicted by continued improvement of FTM scores), he is currently programmed on low stimulation settings. In this patient, the change in stimulation-induced side effects is likely related to increased brain atrophy, as shown across 4 atrophy indices.

Contact	Initial monopolar review* Voltage (Side effect)		Current monopolar review* Voltage (Side effect)		Change in voltage	Change in time (months)

0	0.6 (right hand paresthesia)		0.5 (right hand paresthesia; also right jaw and leg pulling at ~1V)		–0.1	78
1	0.8 (right arm paresthesia)		0.9 (right jaw pulling and right oral paresthesia)		+0.1	
2	2.3 (right face paresthesia)		1.9 (right jaw pulling; also right arm tingling at ~2V)		–0.4	
3	4.5 (right face paresthesia)		2.9 (right arm and jaw paresthesia; also concurrent pulling at ~3.4V)		–1.6	
	**Pre-operative tremor scores**	**1-year tremor scores, after left VIM-DBS**	Current tremor scores**			
FTM	55 (motor); 18 (ADL)	23 (motor, DBS-ON); 37 (motor, DBS-OFF); 6 (ADL)	21 (motor, DBS-ON); 43 (motor, DBS-OFF); 2 (ADL)			
**Change in brain atrophy measurements and lead localizations**^1–3^						
	**Evans index (normal < ~0.3)**	**Bifrontal index (normal < ~0.3)**	**Ventricular index (normal < ~0.3)**	**Cella media index (normal < ~0.25)**		

CT after initial left VIM-DBS (2013)	0.27	0.32	0.31	0.16		
Most recent CT (2019)	0.31	0.38	0.37	0.18		
% change	15%	19%	19%	13%		

* Monopolar threshold reviews were performed with PW 90 Freq 135. ** Most recent tremor scale was performed 7 years after left VIM-DBS implantation; 3 months after activation of right VIM-DBS but 6 years since original right VIM-DBS implantation. Left VIM-DBS lead = contacts 0–3. PW = pulse width (μs); Freq = frequency (Hz); VIM = ventralis intermedius nucleus; DBS = deep brain stimulation; FTM = Fahn-Tolosa-Marin Clinical Rating Scale for Tremor; ADL = activities of daily living.

**Figure 1:**
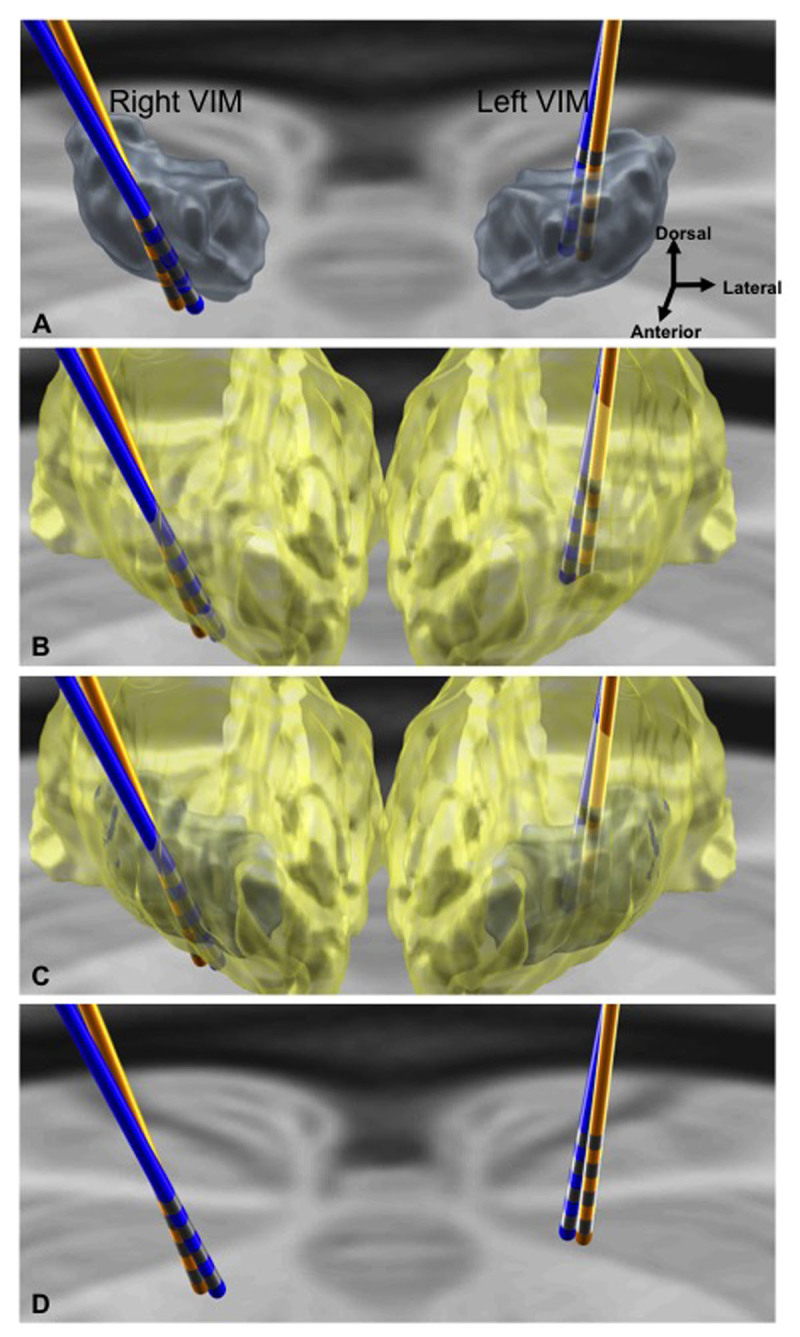
**3D rendering of bilateral DBS VIM electrodes, initial vs. current.** Sections A–C depict bilateral VIM leads in relation to targeted grey structure of VIM proper **(A)**, yellow structure of thalamus **(B)**, and overlay of VIM within the thalamus **(C)**. Section D shows relative lateral shift of current VIM electrodes. The original DBS lead location is depicted in blue, and current DBS lead location in orange. His current optimized DBS settings are: 2- C+1.7V PW 90 Freq 135 (left VIM), and 10- C+ 1.0V PW 90 Freq 135 (right VIM).

**Figure 2:**
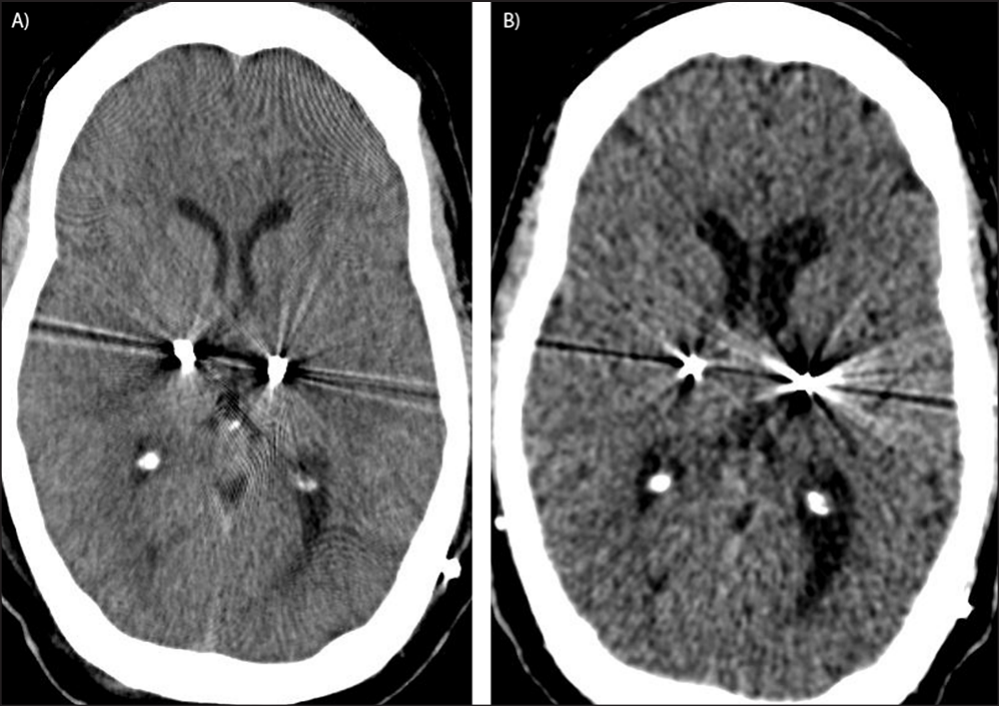
**Changes in noncontrast head CT over time.** Initial postoperative CT in 2014 (**A**) was normal, with bilateral VIM-DBS leads in place. Recent CT in 2019 (**B**) shows increased ventricular size and subtle left > right hemispheric atrophy.

Brain imaging was repeated to reassess lead localization, comparing images from 5 years prior (Figures 1, 2). Ventricular size relative to brain tissue and cerebral atrophy were assessed by applying several atrophy indices, including the 1) Evans index (ratio of the maximal frontal horn ventricular width to the maximal inner diameter of the skull); 2) the bifrontal index (ratio of the maximal frontal horn ventricular width to the inner diameter of the skull in the same plane); 3) the ventricular index (ratio of the minimum width of lateral ventricules to the maximum bifrontal diameter); and 4) the cella media index (ratio of the minimum distance between lateral walls of the lateral ventricles in cella media region to the maximum inner skull diameter) [2,3,4]. Changes in ventricular size and atrophy were present across all indices (Table 1). The changes correlated with a relative lateral shift of both previously implanted DBS leads. The evaluation of brain atrophy and shift in our patient was based on lead location assessment using a modifiable atlas and direct visualization.

Additional radiographic analysis was completed to estimate changes in total brain volume using a CT-based tool, given limitations of patient’s available imaging studies. This confirmed a decrease in total brain volume over time from 1130.5 cm^3^ preoperatively, to 1051.8 cm^3^ post-operatively. CT-based brain volume estimation software is less accurate than MR-based method, but the longitudinal change in brain volume over 6 years, in an otherwise cognitively normal adult, is in keeping with evidence of brain atrophy.

Reprogramming of the DBS leads resulted in sustained clinical benefit, though the reprogramming was at lower than usual ET DBS settings (Supplementary Table 1).

## Clinical dilemma

This case illustrates a patient presenting with a change in the benefit of a long-term implanted VIM-DBS for tremor. There was a narrowing of the therapeutic thresholds across DBS lead contacts. These changes coincided with increased brain atrophy and an overall lateral shift of VIM-DBS leads. The following questions are raised: 1) What is known about the potential causes of worsening tremor and reduced side effect thresholds in chronically implanted VIM-DBS, including the effect(s) of brain atrophy, and 2) What are the programming strategies to manage stimulation-induced complications from atrophy-related lead migration?

## Clinical solution

The patient experienced tremor suppression even with reprogramming at lower electrical stimulation parameters. If reprogramming were unsuccessful, reimplantation may have been necessary. This patient’s particular side effect profile was likely due to current spread into the ventralis caudalis (Vc) nucleus of the thalamus at the ventral contacts (e.g. sensory side effects), and capsular spread (e.g. motor side effects) at the dorsal contacts. The dorsal contacts are closer to the lateral border of the thalamus, and thus neighboring the internal capsule fibers on a lateral to medial trajectory. To avoid capsular side effects, we did not utilize the dorsal-most contact, which, given the angle and trajectory of the VIM-DBS lead, would be located closest to the internal capsule. Bipolar electrode configurations facilitated the concentration of the volume of tissue activated (VTA) in the region of interest within the target. The bipolar programming strategy also helped to avoid the current spread to unintended areas. At subsequent programming visits, the patient was able to tolerate small increments in pulse width (PW), which may have helped to compensate for lower stimulation amplitudes. These programming strategies overall provided sustained improvement in tremor control without inducing intolerable sensorimotor side effects, worsening speech or diminishing gait.

## Gaps in knowledge

### Effects of brain atrophy in chronic DBS

Brain atrophy commonly occurs with aging, and in our patient, the ET itself, the other medical complications, including the history of bipolar disorder (though patient has remained psychiatrically stable, off psychotropic agents), the severe systemic infections, and the acute ischemia all could have collectively contributed to atrophy. While evidence of enlarged ventricles may also raise the possibility of normal pressure hydrocephalus (NPH), our patient did not have the classic triad of clinical symptoms, including gait disturbance, dementia, and urinary incontinence. Although no detailed postsurgical neuropsychological evaluation was conducted at our institution due to multiple complications, patient did not have functional cognitive impairments affecting his activities of daily living (ADLs). Prior research has shown that evaluation of ADLs (e.g., as measured in the Fahn-Tolosa-Marin Clinical Rating Scale for Tremor [FTM]) may be a more sensitive marker for cognitive decline [5,6].

Thalamic volume is known to decline with advancing age [7], but the expected rate of brain atrophy for DBS patients remains unknown. Previous case reports of the subthalamic nucleus (STN) and VIM-DBS described a relative lateral shift of DBS leads due to atrophy [2,8]. This is not actually a lateral movement through brain tissue, but rather a movement of the lead along with the brain tissue. One possible rationale for the lateral shift was the ventricular enlargement resulting from a loss of white matter tissue with aging [8,9]. The atrophy and lateral shift have been shown in scattered reports to narrow long-term programming therapeutic windows [2]. Further studies are needed to quantify atrophy and measure changes in the VTA, concurrent with changes in lead location.

### Other potential factors affecting long-term DBS efficacy and changes in stimulation-induced side effects

While VIM-DBS is a safe and effective therapy for patients with severe refractory ET, up to 40–70% of patients will experience worsening of tremor over time [10,11]. Potential causative factors include disease progression, disruption of oscillatory neuronal pathways, side effects from long-term stimulation, changes in impedance, tolerance, or a combination thereof [2,12,13,14]. Stimulation of structures closer to the midline such as the thalamus and STN, can manifest ipsilateral benefits and side effects. Accordingly, the activation of both channels may lead to narrower therapeutic windows compared to testing each electrode independently, and some reprogramming may thus be necessary. This phenomenon is rare and was not observed in our patient.

For our patient, disease progression may play a role but unlikely to be the sole cause, as our patient’s FTM motor scores indicated continued benefit with chronic stimulation over time. The imaging that revealed an increase in atrophy greater than expected from age alone was a compelling finding. While some attribute tolerance (i.e., progressive loss of benefit to stimulation or habituation) to diminished tremor suppression, tolerance has been over-reported in the literature [12]. If habituation were at play, we should not see a change of side effect thresholds at all contacts, as seen with our patient. The appearance of sensory side effects at lower voltages would also suggest a different cause. Tolerance was not demonstrated in our case, given the robust response to programming initially and the immediate response to reprogramming.

Another potential explanation for changes in long-term DBS effectiveness was impedance variability. Impedance can affect the VTA and thus impact clinical effectiveness. In our case, impedance changes were difficult to assess as the patient 1) underwent replacement of new dual-channel IPG; and 2) his initial single-channel IPG reached end-of-battery life several years prior, making it impossible to interrogate and to ascertain fair comparisons. While some studies observed random changes in long-term impedance [15], others showed a continued decline of impedance over time despite stable stimulation parameters [16]. This decline in impedance may lead to consequent enlargement of the local VTA. Clinicians should be aware of this potential phenomenon of chronic DBS, though there is scant literature to suggest any impact on outcome. Notably, the clinician programmer can opt to switch to a constant current mode to avoid issues with impedance changes, but this approach may improve symptoms at the expense of premature battery drain.

Finally, with loss of benefit, it is always important to assess for hardware-related complications, e.g. DBS lead/wire break. The reported incidence of this complication is approximately 5% [17,18]. Hardware-related issues were not encountered in our patient.

### Management of changes in clinical effectiveness and threshold tolerances in chronic VIM-DBS

Managing neurostimulation parameters in VIM-DBS has been limited, empirical, and primarily based on expert opinion. Generally, the initial approach is to avoid unmasking the presence of cerebellar features, which may result from excessive stimulation into unintended tracts. One strategy has been the reduction of current density by reducing PW or the amplitude of stimulation. After discovering the threshold for stimulation-induced cerebellar tremor or ataxia, the frequency and amplitudes can be slowly incremented until the tremor is controlled or intolerable adverse effects manifest [14]. The advent of directional DBS may possibly address these stimulation-induced side effects, which may potentially be avoided by delivering stimulation in an axially asymmetric fashion, away from unintended tracts. With newer technologies, clinicians also have the flexibility of exploring voltage-controlled or current-controlled stimulation. In our patient, voltage-controlled programming was the only one used throughout. Stimulation parameters can be initially fixed by the clinician, commonly PW of 60 μsec and a frequency of 130 Hz during this “threshold discovery” process. Paresthesia in VIM-DBS is perhaps the most common transient side effect from the electrical field reaching the Vc region, which is posterior to the VIM. Sensory issues typically resolve with reducing stimulation [19,20]. Speech impairment is another common complaint, especially following bilateral VIM-DBS. It is critical to determine if dysarthria is due to disruption of cerebellar-thalamic fibers more ventrally, or from the impact on motor fibers of the internal capsule more laterally. Knowing these relationships can guide activation of appropriate DBS contact(s). Depending on side effects, other programming configurations might be utilized in an effort to shape the VTA and may include bipolar lead configurations, reversing the polarity of an existing bipolar setting, interleaved stimulation or adding stimulation by adding another contact (e.g. using the most ventral contact if possible). Other suggested strategies include turning off the DBS at night [21], using DBS holidays, or activating the DBS only when needed (e.g. eating) [14]. Lead revision or rescue may sometimes be necessary when optimized programming fails to resolve the issue. In our patient, a repeat neuropsychological evaluation would be critical if future surgical candidacy was being considered. The considerable brain atrophy and other medical conditions present in our patient would need to be carefully weighed.

Our case highlights a potential complication encountered in long-term VIM-DBS in a patient with worsening clinical outcome and side effects at lower electrical stimulation thresholds. Brain atrophy should be assessed in these cases and reprogramming attempted, prior to consideration of a repeat surgical intervention. Future studies will be needed to further characterize changes in the VTA associated with atrophy, as well as to assess the impact of new technologies (e.g. current steering) on this phenomenon. One might say that management of brain atrophy following DBS is an exercise in following a moving target.

## Expert Commentary

This case report reviews a commonly encountered challenge in managing ET with long term VIM-DBS – worsening tremor over time – and highlights brain atrophy and changes in the measured lead position as a possibly under-appreciated etiology of loss of therapy benefit with narrowing of the therapeutic window. The paper provides a useful checklist for clinician programmers to consider when troubleshooting patients with worsening ET tremor.

Accurate targeting of the intended brain structure is vital for maximizing benefits. Recent and future technological advances in DBS lead and IPG technology may facilitate greater programmer adaptability for suboptimally placed electrodes, regardless of the etiology. Radially segmented electrodes have been designed to steer DBS current axially toward the therapy target and to shape the stimulation. Current fractionalization, whether through a multi-stim set (“interleaving”) or multiple independent current control, can facilitate customizability of the activated tissue. Leads with greater contact numbers and longer spans may add vertical expansion. Programming interfaces which model the volume of tissue activated may assist in troubleshooting. Future electrophysiological biomarkers to guide closed-loop stimulation may further customize DBS. Collectively, these technologies represent opportunities to “follow a moving target” without more invasive surgery. Future studies on the relative efficacy of these strategies, their implications on power drain, and their ease of use will be needed.

## Supplementary File

**Supplementary Table 1:** DBS stimulation parameters.

DBS lead	Initial programming after monopolar review of left VIM-DBS	1-year after left VIM-DBS, optimized programming	Current optimized programming, after new IPG

Left VIM-DBS	2- C+ 2.0v PW 90 Freq 180	2- C+ 2.5v PW 120 Freq 185	2- C+ 1.7v PW 90 Freq 135
Impedance	1412	1277	770
Right VIM-DBS	N/A	N/A	10- C+ 1.1v PW 90 Freq 135
Impedance	N/A	N/A	813

PW = pulse width (μs); Freq = frequency (Hz); Impedance = Ohms (Ω); VIM = ventralis intermedius nucleus; DBS = deep brain stimulation; IPG = implantable pulse generator.
